# Temporal Persistence of the Biological Effects of a Seed-Applied Biostimulant on Wheat (*Triticum aestivum*) Germination and Early Growth Under Extended Pre-Germination Soil Drought

**DOI:** 10.3390/plants15172552

**Published:** 2026-08-22

**Authors:** Diana Elena Bolohan, Maria Apostol, Lucian Raus

**Affiliations:** 1 Department of Pedotechnics, Faculty of Agriculture, “Ion Ionescu de la Brad” Iasi University of Life Sciences, 3 Mihail Sadoveanu Alley, 700490 Iasi, Romania; diana.bolohan@iuls.ro; 2 Department of Horticultural Technologies, Faculty of Horticulture, “Ion Ionescu de la Brad” Iasi University of Life Sciences, 3 Mihail Sadoveanu Alley, 700490 Iasi, Romania; maria.apostol@iuls.ro

**Keywords:** *Triticum aestivum*, seed dressing, triticonazole, delayed post-sowing watering, dry sowing conditions, seminal roots

## Abstract

Delayed post-sowing watering may leave treated wheat (*Triticum aestivum*) seeds in dry soil for several weeks before germination begins, raising the question of how long the effects of seed-applied biostimulants remain biologically active under these conditions. This study evaluated the persistence of the biological effects of a seed-applied biostimulant, applied alone (BS) or combined with a triticonazole-based fungicide (TBS), after delayed watering of wheat seeds. Seeds were watered immediately after sowing or after 2, 3, 4, or 5 weeks and evaluated in Petri dishes and growth pots. Compared with the untreated control, BS and TBS reduced mean germination time by up to 9.4 h and 8.7 h, respectively (*p* < 0.05), while also promoting greater seminal root development after prolonged exposure to dry conditions. In the pot experiment, treatments including the biostimulant maintained greater root dry weight at both BBCH 11 and BBCH 12, indicating that a measurable stimulatory response was still observed beyond germination and early seedling establishment. The strongest responses were observed when watering was delayed by 2–3 weeks, remained evident after 4 weeks, and declined after 5 weeks of dry sowing conditions. Under the conditions of this experiment, the results demonstrate that, although the biostimulant supports early root system development after moisture restoration, the magnitude of the treatment response is conditioned by the duration of the pre-germination waiting period.

## 1. Introduction

Winter wheat (*Triticum aestivum* L.) is a critical global crop, requiring rapid establishment and uniform emergence for adequate pre-winter development. In temperate continental climates, wheat is typically sown between September and October. However, frequent autumn precipitation deficits can leave seeds inactive in dry soil for several weeks before germination. While conservation tillage can help preserve seedbed moisture, successful crop establishment ultimately depends on the seeds’ ability to maintain their vigor during this prolonged dry period until water becomes available [1,2,3,4,5,6].

Wheat seeds can survive severe drought conditions without losing their capacity to germinate. This ability is supported by internal protection systems and cellular repair mechanisms that become active as soon as the grain absorbs water [3,7,8]. However, the maintenance of viability is not equivalent to the maintenance of vigor. After prolonged periods in dry soil, seeds may require more time to germinate, exhibit less uniform germination, and produce seedlings with weaker initial growth. Therefore, the final germination percentage alone is insufficient to assess seed performance. Indicators such as mean germination time, seed vigor index, radicle length, and coleoptile length provide a clearer assessment of the ability of seeds to rapidly initiate growth processes after moisture is restored [2,4].

In this context, seed treatments play an important role in protecting and supporting the early stages of plant development. Fungicides used for seed treatment provide phytosanitary protection against seed-borne and soil-borne pathogens, thereby contributing to the maintenance of seed viability. However, some compounds belonging to the triazole group may also exert physiological effects on plants by interfering with gibberellin biosynthesis and cell elongation processes [9,10,11]. In recent years, biostimulants have increasingly been used as seed treatments to support the physiological processes involved in early seedling growth, particularly under various abiotic stress conditions. Recent studies in wheat have further shown that seed-applied or microbial biostimulants can modify plant responses to water deficit, including early growth, root development, and physiological responses under drought conditions [12,13]. They may supply nutrients in the immediate vicinity of the seed, but they are primarily applied to stimulate hormonal and biochemical processes that may improve plant growth under unfavorable conditions [14,15,16]. These biostimulants are available in different formulations based on humic and fulvic acids, plant extracts, bioactive compounds and micronutrients. In this study, Oligal SD—a commercial biostimulant formulated from a blend of manganese, zinc, boron, and an organic complex of humic and fulvic acids—was evaluated. Humic and fulvic substances are known to modify cell membrane permeability and improve water binding capacity within seed tissues during early imbibition, facilitating smoother rehydration. The inclusion of zinc and manganese directly supports the activation of hydrolytic enzymes and antioxidant defense systems, which are essential for maintaining embryo vigor and accelerating metabolic repair processes when hydration of the seeds is delayed [17,18,19,20]. Direct application to the seed may enhance the biological response because these bioactive compounds remain in close proximity to the plant at germination, before the root system becomes fully functional.

Rapid radicle development is a key factor for winter wheat during the earliest stages of growth. Enhanced early seminal root initiation favors the early exploration of the substrate within the immediate rhizosphere, water uptake, and access to nutrients, which is particularly important in regions where germination is delayed by limited water availability [21,22,23]. In addition to morphological parameters, biomass accumulation and nitrogen concentration can provide functional information on seedling status during the early stages of growth. Nitrogen supports photosynthesis and the growth of young tissues, while its accumulation in roots and shoots reflects both nutrient availability in the soil and the capacity of the root system to support plant growth after emergence [24,25,26]. However, plant nitrogen values within the optimal range should be interpreted together with biomass because greater nitrogen accumulation may reflect more vigorous growth, greater nitrogen acquisition, or a combination of both processes [27].

The effects of biostimulants on germination, root development, and plant tolerance to abiotic stress have been widely discussed in the literature. However, it remains unclear how long the biological effects of a seed-applied biostimulant remain detectable when treated seeds are sown in dry soil. Moreover, it is unknown whether prolonged exposure to dry conditions before germination affects the subsequent expression of these effects on plant growth and development. Most studies evaluate seed or seedling responses under conditions of immediate hydration or under water stress imposed after germination. By contrast, relatively few studies have examined the practical situation in which treated seeds are sown in dry soil and remain physiologically inactive for several weeks. This time window is highly relevant for autumn-sown crops; as orthodox seeds, wheat grains do not enter a state of true physiological dormancy under moisture deficits but rather a state of metabolic quiescence. Even in this inactive state, the seeds are not static but remain susceptible to physiological changes associated with prolonged exposure to dry soil conditions, which may affect subsequent seedling vigor [28,29].

Therefore, this study aimed to determine the exact time window (ranging from 2 to 5 weeks of dry sowing) during which a seed-applied biostimulant, used either alone or in combination with a triticonazole-based fungicide, continues to provide measurable biological effects on wheat germination, early root development, and seedling nitrogen accumulation upon subsequent hydration.

We tested the hypothesis that prolonged exposure of treated seeds to dry soil conditions would progressively attenuate the biological effects of the seed-applied biostimulant, while the combination with the fungicide could modify the duration and magnitude of these effects by limiting pathogen-related damage and potentially supporting early root performance in the presence of triticonazole.

## 2. Results

### 2.1. Germination Parameters and Seedling Vigor in Petri Dishes

Germination parameters and seedling vigor were evaluated under laboratory conditions in Petri dishes to determine the effects of the applied treatments on germination and the initial growth of wheat seedlings.

The seed germination percentage ranged from 94 to 97% across all watering regimes (W1–W5). Differences among treatments did not exceed 3% and were not statistically significant (*p* > 0.05). The lowest germination percentage was recorded in the untreated control (C). The treated variants showed slightly higher but comparable values. These results indicate that the treatments did not affect the final germination capacity of the seeds under the experimental conditions, even when watering was delayed for up to five weeks (Table S1).

**Figure 1:**
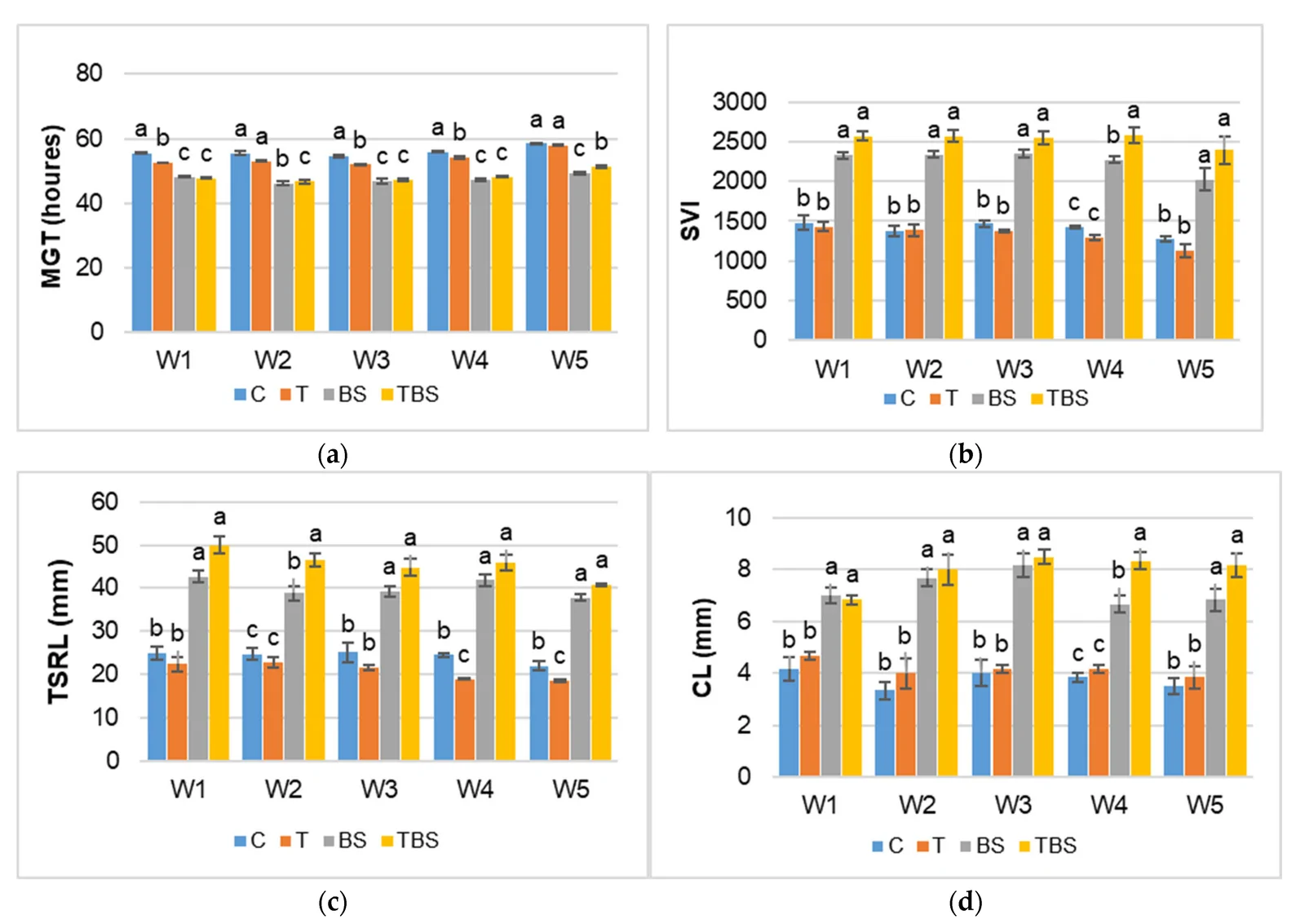
(**a**) Mean germination time (MGT), (**b**) Seed vigor index (SVI), (**c**) Total seminal root length (TSRL) and (**d**) coleoptile length (CL) of wheat (*Triticum aestivum*) seedlings grown in Petri dishes under different seed treatments and watering regimes. Bars represent mean values ± standard error (SE). Different letters indicate statistically significant differences among seed treatments within each watering regime according to Tukey’s test (*p* < 0.05). Letters should not be compared across watering regimes. Abbreviations: C—Control; T—Triticonazole; BS—Biostimulant; TBS—Triticonazole + Biostimulant. W1–W5—watering intervals.

Mean germination time (MGT) values ranged from 47.7 to 55.5 h under immediate watering (W1). The untreated control (C) had an MGT approximately 3 h longer than the fungicide treatment (T). Conversely, biostimulant application significantly reduced MGT by 7.3 h (BS) and 7.8 h (TBS). Under the two-week watering delay (W2), C and T variants showed similar values of 55.5 and 53.2 h. However, biostimulant treatments significantly accelerated germination, reducing MGT by 9.4 h in BS and 8.7 h in TBS. Extending the dry period to three weeks (W3) resulted in an MGT of 54.5 h for the control group. Compared to C, MGT decreased by 3.3 h in T, 7.6 h in BS, and 7.1 h in TBS. A matching temporal trend occurred at the four-week delay (W4), with BS and TBS maintaining the lowest values. At the longest watering delay (W5), germination times increased slightly for C (58.7 h) and T (57.2 h). In contrast, biostimulant treatments significantly decreased MGT to 49.2 h (BS) and 50.8 h (TBS) (Table S1, Figure 1a).

Seed Vigor Index (SVI) values ranged between 1379 and 1433 for C and T groups under early watering (W1–W2). Conversely, SVI increased significantly from 2332 to 2572 in biostimulant-treated seedlings (BS and TBS). Figure 1b illustrates these higher values. As the pre-watering interval extended from W3 to W5, the vigor index in T decreased slightly. This parameter reached its minimum of 1125 at W5. When applied alone (BS), the biostimulant showed a moderate SVI reduction at W4 and W5. Nevertheless, values remained significantly higher than C and T. The combined TBS treatment maintained stable, consistently high vigor index values throughout the experiment. Even under the longest watering delay (W5), the minimum TBS value (2399) exceeded the maximum values of C and T under immediate or early watering (Table S1).

Overall, a clear temporal trend was observed across the watering regimes: the acceleration of germination (MGT) and enhancement in seedling vigor (SVI) by the biostimulant treatments reached their maximum expression under moderate watering delays (W2–W3), followed by a progressive decline in the magnitude of this benefit as the dry period extended to W5.

### 2.2. Biometric Parameters of Seedlings

Total seminal root length (TSRL) was calculated as the sum of the lengths of the three seminal roots. Biostimulant application resulted in significantly greater TSRL values, reaching 42.7 mm in BS and 50.0 mm in TBS, compared with 25.0 mm in the untreated control. The fungicide treatment (T) produced a slightly lower value of 22.3 mm. When watering was delayed by two weeks (W2), values for C and T remained similar to those observed under W1 and belonged to the same statistical group. By contrast, the combined treatment, TBS, maintained a significantly greater TSRL value of 46.5 mm (*p* < 0.05). Under the three-week watering delay (W3), values ranged from 21.5 mm in T to 44.7 mm in TBS. After a four-week watering delay (W4), the untreated control maintained a relatively stable TSRL value of 24.4 mm, whereas the fungicide treatment resulted in a significant reduction to 18.9 mm. In contrast, the biostimulant-treated variants showed higher values of 41.8 mm in BS and 46.0 mm in TBS. Under the longest watering delay (W5), all variants showed a slight reduction in root growth, with values ranging from 18.5 mm in T to 40.7 mm in TBS. Nevertheless, the biostimulant treatments maintained significantly higher values than the untreated control (Table S1).

This general pattern is also shown in Figure 1c, with greater TSRL values in the biostimulant-treated variants and lower values in the variant treated with the fungicide alone.

Coleoptile length (CL) was significantly greater in the BS and TBS treatments due to the application of the biostimulant, reaching values approximately twice as high as those recorded for untreated seeds or seeds treated with the fungicide alone. This effect was maintained across all watering regimes simulated in the Petri dishes (Figure 1d). Coleoptile length in the untreated control ranged from 3.3 to 4.2 mm and remained among the lowest values irrespective of the watering regime. The fungicide treatment did not result in significant differences relative to the control, with coleoptile length ranging from 3.8 to 4.7 mm and reaching its maximum under immediate watering (W1). Biostimulant application alone resulted in greater coleoptile length under the first three watering regimes, with values ranging from 7.0 to 8.2 mm. Slightly lower values of 6.7–6.8 mm were recorded under the longer watering delays, W4 and W5. The combined TBS treatment maximized coleoptile length under W2–W5, maintaining values between 8.0 and 8.5 mm. Conversely, immediate watering (W1) resulted in a lower biological response of 6.8 mm (Table S1). In summary, initial seedling elongation showed a clear temporal pattern in the treatment responses. The biostimulant variants consistently maintained significantly higher morphological values, mitigating the progressive growth constraints caused by extended pre-germinative drought.

### 2.3. Emergence and Morphological Development of Seedlings in Pots

**Figure 2:**
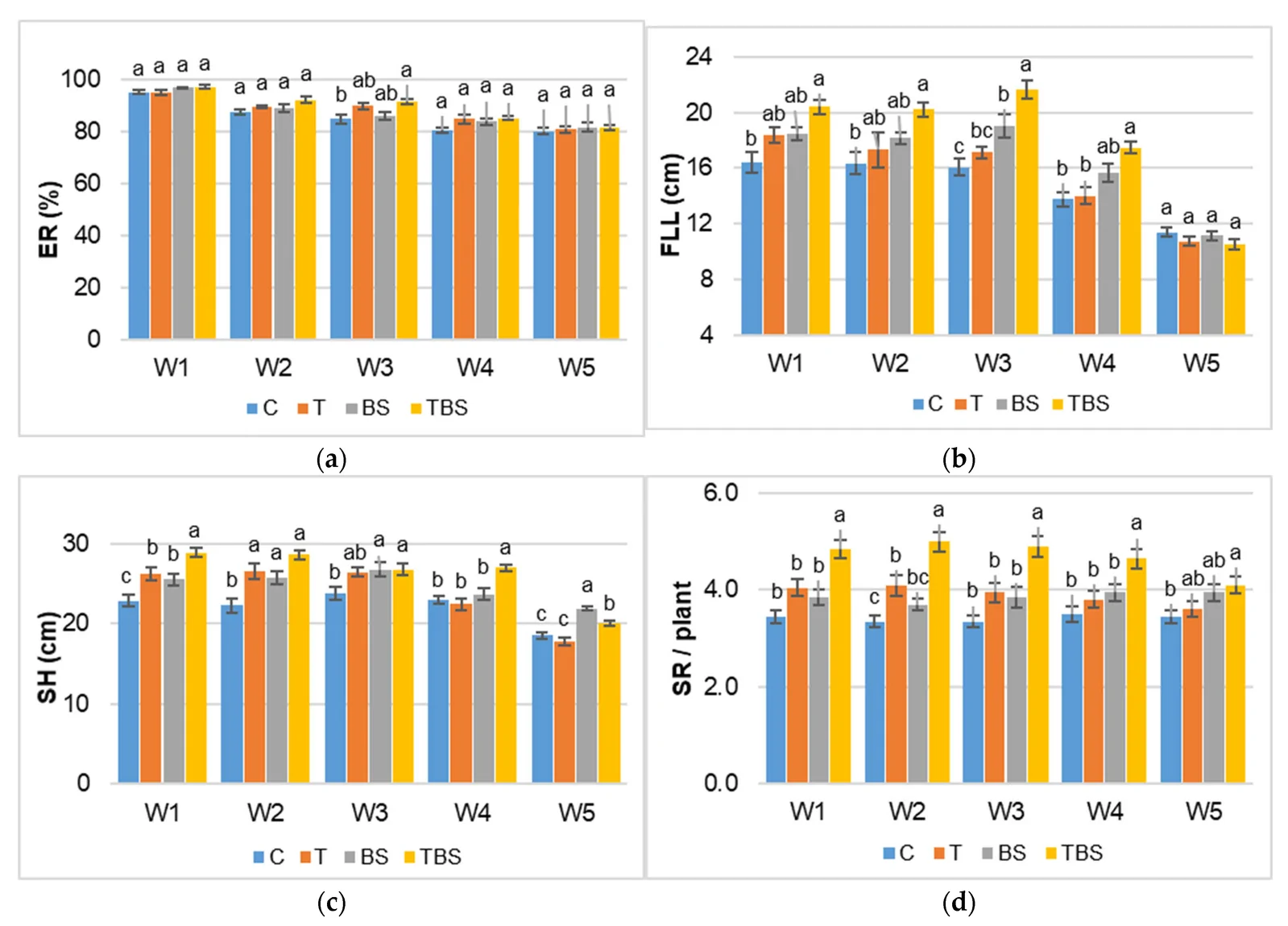
(**a**) Emergence rate (ER), (**b**) First leaf length (FLL), (**c**) Shoot height (SH) and (**d**) Seminal root number (SR) of wheat (*Triticum aestivum*) seedlings grown in pots under different seed treatments and watering regimes. Bars represent mean values ± standard error (SE). Different letters indicate statistically significant differences among seed treatments within each watering regime according to Tukey’s test (*p* < 0.05). Letters should not be compared across watering regimes. Abbreviations: C—Control; T—Triticonazole; BS—Biostimulant; TBS—Triticonazole + Biostimulant. W1–W5—watering intervals.

Emergence rate (ER) values ranged from 95% to 97% under early water availability (W1) without significant treatment-induced differences; however, this parameter experienced a notable reduction to 87.3–92.0% following a two-week delay in watering (W2). The greatest value was recorded for TBS, although it did not differ significantly from the other treatments. After three weeks without watering (W3), significant differences among treatments became evident, with the largest difference, 5.6 percentage points, occurring between TBS and the untreated control. When watering was delayed by four weeks (W4), emergence was lower in the control (80.3%) and higher in the treated variants, reaching 84.7% in T and 85.0% in TBS. Delaying watering for five weeks (W5) resulted in similar emergence rates among variants, ranging from 80.0 to 81.3%, with no significant differences among treatments (Figure 2a).

To evaluate the influence of the treatments on early seedling development, the main biometric parameters were assessed at the BBCH 12 growth stage: first leaf length, leaf sheath length, shoot height, and seminal root number. Mean values ± standard error are presented in Table S2.

First leaf length (FLL) exhibited significant differences between the untreated control and the TBS treatment under immediate watering (W1) and a two-week watering delay (W2), reaching values of 16.4 cm and 20.4 cm, respectively. This biostimulant-induced variation became even more pronounced when watering was delayed by three weeks (W3). Under this regime, significant differences were recorded among C, BS, and TBS, with mean values of 16.1, 19.1, and 21.7 cm, respectively. When the watering delay was extended to four weeks (W4), first leaf length decreased slightly, with values ranging from 13.8 to 17.5 cm. Tukey’s post hoc test indicated that C and T belonged to the same statistical group and had significantly lower values than TBS. Under W5, no significant differences were detected among treatments, with mean values ranging from 10.5 to 11.4 cm.

The lowest values were recorded in the variants that included the fungicide, T and TBS (Table S2). Figure 2b shows the higher first leaf length recorded for the combined treatment, with a maximum under W3. Beginning with W4, first leaf length decreased in all variants, and under W5 the values became similar among treatments.

Mean shoot height (SH) reached its maximum value of 28.9 cm in the TBS treatment under early water availability (W1), compared to a minimum of 22.8 cm recorded in the untreated control (C). The differences were statistically significant, and the treatments were assigned to three distinct statistical groups: C (c), T and BS (b), and TBS (a). When watering was delayed by two weeks (W2), shoot height remained at a similar level, although significant differences were detected between the untreated control and the other treatments. Values ranged from 22.2 cm in C to 28.6 cm in TBS. Under a three-week watering delay (W3), treated variants (T, BS, TBS) exhibited similar shoot heights (26.4–26.7 cm), whereas the untreated control (C) showed lower values (23.8 cm). Extending the drought to four weeks (W4) resulted in convergent shoot height values between 22.4 and 23.7 cm across C, T, and BS treatments. The combined treatment maintained a greater shoot height of 27.0 cm and differed significantly from the other variants. Under the longest watering delay (W5), significant differences among treatments were observed, and the treatments were assigned to three statistical groups. No relevant difference was recorded between the control and the fungicide treatment, with T showing the lowest value. The greatest shoot height was recorded in BS at 21.9 cm, followed by TBS at 20.0 cm (Table S2; Figure 2c).

The number of seminal roots (SR) was consistently the lowest in the untreated control, where it averaged between 3.35 and 3.50 roots plant^−1^ across all watering regimes. The fungicide treatment increased the number of seminal roots under all watering regimes relative to the untreated control. The largest difference, 0.75 roots plant^−1^, was observed under W2 and was statistically significant. The difference associated with the fungicide treatment gradually decreased as the watering delay increased, reaching 0.25 roots plant^−1^ relative to the control under W5. Plants treated with the biostimulant alone showed small increases of 0.35–0.50 roots plant^−1^ relative to the control, although the differences were not statistically significant under all watering regimes. The greatest values were recorded for the combined treatment, with a maximum of 5 roots plant^−1^ under W2 (Table S2). The graphical representation shows significant differences between TBS and the other treatments under W1–W4. Under the longest watering delay (W5), the treatment effect was reduced, and a significant difference was maintained only between TBS and the untreated control (Figure 2d).

Conclusively, shoot height and leaf development exhibited a coordinated temporal decline. The numerical advantage of the combined TBS treatment was maximized under moderate stress progressively declined with prolonged dry sowing.

### 2.4. Seedling Biomass Accumulation

Root and shoot dry weight at the two growth stages provided information on the effects of the seed treatments on biomass accumulation in the root system and shoots.

Root dry weight (RDW) at BBCH 11 was significantly influenced by the seed treatments. However, numerical differences among variants remained relatively small (Table S3). Under immediate watering (W1), the untreated control (C) recorded the lowest value of 11.1 mg plant^−1^. In contrast, biostimulant treatments (BS and TBS) achieved the highest dry weights, reaching 12.7 and 12.8 mg plant^−1^. Both treated variants differed significantly from the control group. A similar response was observed when watering was delayed by two weeks (W2), although only TBS differed significantly from the control. Under the three-week watering delay (W3), the greatest values were recorded in the variants that included the fungicide, T and TBS. After a four-week watering delay (W4), differences among treatments were no longer statistically significant. Under the longest delay (W5), T showed the lowest root dry weight, at 9.0 mg plant^−1^, whereas BS produced the greatest value, at 12.2 mg plant^−1^. The difference was statistically significant.

**Figure 3:**
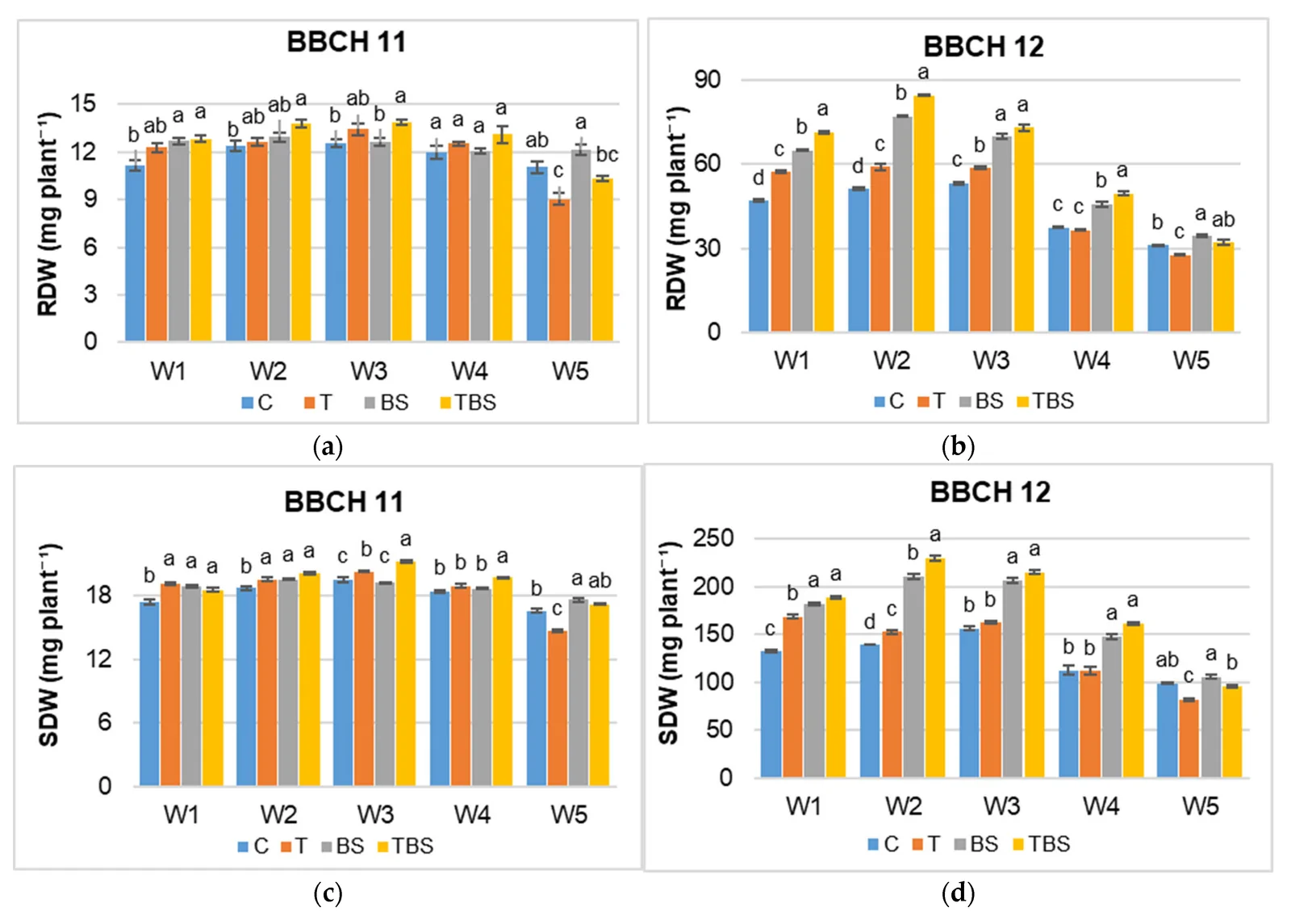
Effects of seed treatments under five watering regimes on root dry weight (RDW) of wheat (*Triticum aestivum*) seedlings at BBCH 11 (**a**) and BBCH 12 (**b**) and shoot dry weight (SDW) at BBCH 11 (**c**) and BBCH 12 (**d**). Bars represent mean values ± standard error (SE). Different letters indicate statistically significant differences among seed treatments within each watering regime according to Tukey’s test (*p* < 0.05). Letters should not be compared across watering regimes. Abbreviations: C—Control; T—Triticonazole; BS—Biostimulant; TBS—Triticonazole + Biostimulant. W1–W5—watering intervals.

At BBCH 12, the effects of the treatments on root dry weight became more pronounced (Table S3). Under all watering regimes, the biostimulant-treated variants (BS and TBS) generally showed the greatest values. Conversely, C and, in most cases, T showed the lowest values. Under W1 and W2, all treatments belonged to distinct statistical groups. These variants showed a progressive increase in root dry weight across the treatments. When watering was delayed by three weeks (W3), BS and TBS produced the greatest values of 69.9 and 73.1 mg plant^−1^. No significant difference was detected between them. Under W4 and W5, root dry weight decreased in all variants. Nevertheless, the biostimulant treatments maintained significantly greater values than the control. Their values were also particularly greater than the fungicide treatment. Under W5, BS maintained a relatively high root dry weight. Meanwhile, TBS showed an intermediate response. Its value was significantly greater than T but did not differ significantly from the untreated control. At BBCH 11, Figure 3a shows significant but relatively small differences among treatments under W1–W3. At BBCH 12, the differences became more pronounced as seedling growth progressed. Consequently, BS and TBS showed the greatest root biomass values, particularly under W2 and W3 (Figure 3b).

At BBCH 11, shoot dry weight (SDW) was influenced by the seed treatments, although the differences among variants were smaller than those observed at BBCH 12 (Table S3). Seeds supplied with water immediately after sowing accumulated between 17.5 and 19.1 mg plant^−1^ of shoot dry matter by BBCH 11. All treated variants differed significantly from the untreated control, and the greatest shoot dry weight was recorded for the triticonazole-based fungicide treatment. Seeds supplied with water two weeks after sowing (W2) showed slightly greater shoot dry weight values, and the differences relative to the control were maintained. The combined treatment produced the greatest value, at 20.1 mg plant^−1^. Under W3, TBS maintained the greatest shoot dry weight, at 21.2 mg plant^−1^, followed by T. Under W4, differences among treatments decreased, with TBS remaining the only variant with significantly greater values. Watering after five weeks (W5) resulted in a reduction in shoot biomass in all variants. The lowest value was recorded for T, which was 1.9 mg plant^−1^ lower than the untreated control. The greatest value was recorded when the biostimulant was applied alone (Table S3).

At BBCH 12, differences among treatments became more evident (Table S3). Under immediate watering (W1), all treatments significantly increased shoot dry weight (SDW) relative to the untreated control. The biostimulant-treated variants achieved the highest values, peaking at 188.8 mg plant^−1^ in TBS. A matching trend occurred under W2–W4. Here, BS and TBS consistently maintained significantly greater dry matter than the control. However, shoot dry weight decreased slightly under W4. Under the longest watering delay (W5), all variants showed a marked reduction in shoot biomass. The fungicide treatment (T) recorded the lowest value of 82.4 mg plant^−1^. In contrast, BS maintained the maximum shoot dry weight at 105.4 mg plant^−1^. TBS showed an intermediate response between T and BS. Figure 3c,d illustrate these dynamics. The largest treatment differences appeared under W2 and W3, followed by smaller gaps under W4. Under W5, T showed the minimum shoot dry weight, while TBS values remained higher than T.

The root-to-shoot ratio (R/S) at BBCH 11 and BBCH 12 did not differ significantly among treatments. All variants belonged to the same statistical group. At BBCH 11, R/S values were relatively constant, ranging from 0.62 to 0.69. Conversely, the BBCH 12 ratio was lower, ranging from 0.30 to 0.38. These lower values align with a faster relative increase in shoot biomass compared to root dry matter between the two stages.

Evaluating dry matter accumulation over time revealed a consistent descriptive pattern. The biomass benefits observed at BBCH 11 became more pronounced at BBCH 12, before being significantly constrained by the five-week drought.

### 2.5. Nitrogen Concentration and Accumulation in Wheat Seedlings

At BBCH 12, nitrogen concentration and calculated nitrogen accumulation were assessed separately in roots and shoots to characterize nitrogen distribution within the seedlings.

#### 2.5.1. Nitrogen in Roots

**Figure 4:**
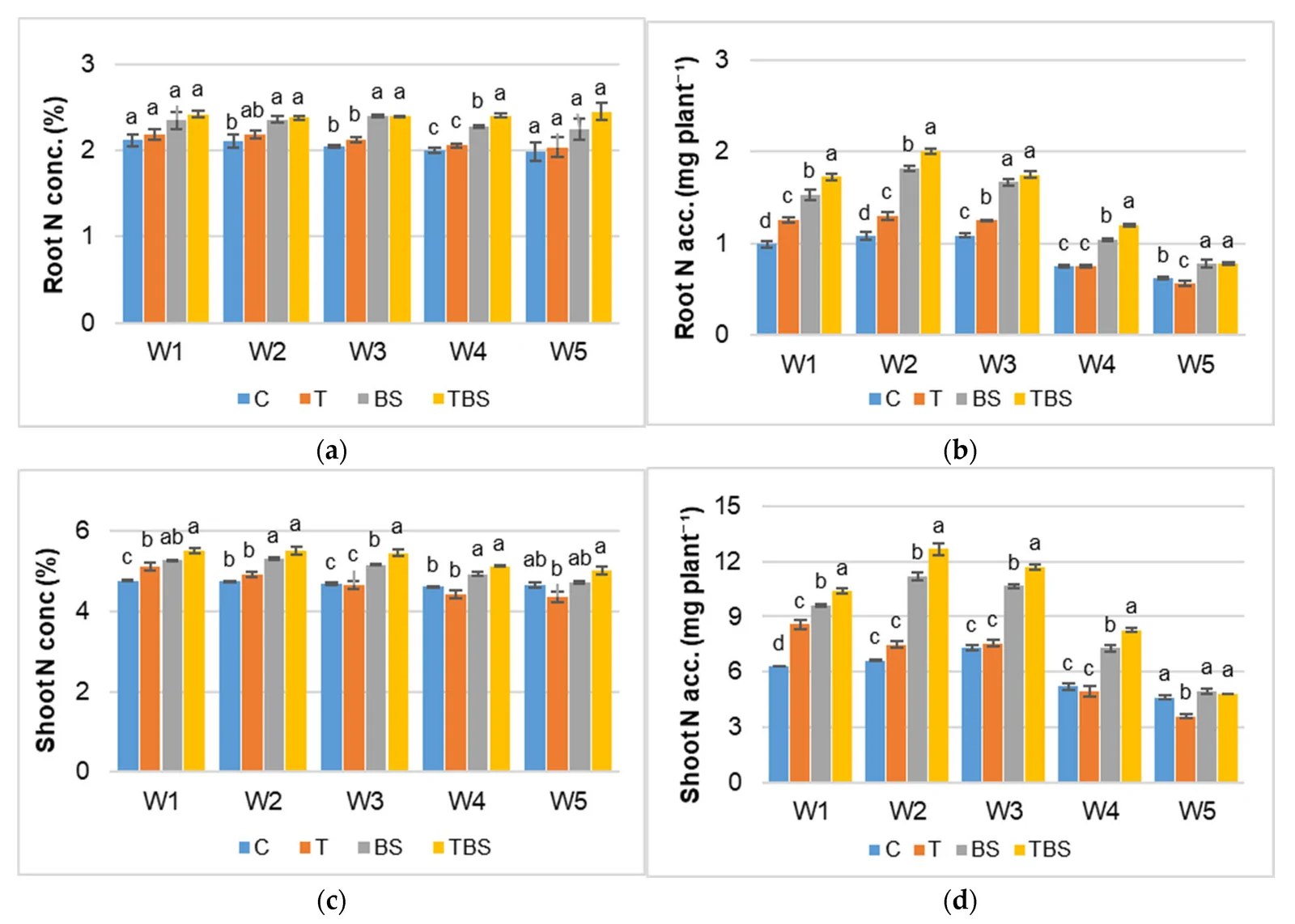
Effects of wheat (*Triticum aestivum*) seed treatments under five watering regimes (W1–W5) on root nitrogen concentration (**a**), root nitrogen accumulation (**b**), shoot nitrogen concentration (**c**), and shoot nitrogen accumulation (**d**) at BBCH 12. Bars represent mean values ± standard error (SE). Different letters indicate statistically significant differences among seed treatments within each watering regime according to Tukey’s test (*p* < 0.05). Letters should not be compared across watering regimes. Abbreviations: C—Control; T—Triticonazole; BS—Biostimulant; TBS—Triticonazole + Biostimulant; W1–W5—watering intervals.

Under W1, root nitrogen concentration ranged from 2.12 to 2.42% N. No significant differences occurred among treatments. Conversely, significant variations emerged under W2–W4. The combined TBS treatment achieved the highest values at 2.38–2.40% N. In contrast, the untreated control (C) recorded the lowest levels at 2.01–2.11% N. Extending the dry window to W5 eliminated these statistical differences (Table S4). Calculated root nitrogen accumulation (mg plant^−1^) differed significantly under W1 and W2. Here, all treatments belonged to distinct statistical groups (a–d), ranging from 0.99 to 2.01 mg plant^−1^. At W3, BS and TBS showed comparable accumulation values of 1.67 and 1.75 mg plant^−1^. Meanwhile, the control group recorded the minimum value of 1.09 mg plant^−1^. Under W4 and W5, root nitrogen accumulation decreased concurrently with root dry weight. Nonetheless, TBS consistently maintained the highest values, differing significantly from all other variants (Table S4). Figure 4a,b illustrate these diverging responses. Nitrogen concentration varied significantly only under W2–W4. However, calculated accumulation differed significantly throughout W1–W5, with BS and TBS consistently achieving the highest levels.

#### 2.5.2. Nitrogen in Shoots

Shoot nitrogen concentration was influenced by the seed treatments, and the magnitude of the differences varied among watering regimes. Under W1–W3, significantly greater nitrogen concentrations were recorded in the treated variants. The greatest value was observed in TBS, reaching up to 5.51% N, followed by BS, whereas C and T showed lower values. Under W4 and W5, differences among treatments became smaller, although TBS maintained the greatest nitrogen concentrations, at 5.12 and 5.02% N, respectively. Under W5, T had a lower nitrogen concentration of 4.35% N compared with 4.65% N in the untreated control.

Calculated shoot nitrogen accumulation differed significantly among variants throughout the watering regimes. Under W1–W3, the greatest values were recorded in TBS, ranging from 10.39 to 12.67 mg plant^−1^, followed by BS. The untreated control showed the lowest accumulation values, ranging from 6.30 to 7.31 mg plant^−1^. Under W4, calculated shoot nitrogen accumulation decreased in all variants, although values remained significantly greater in BS and TBS. Under W5, significant differences were particularly evident for T, which showed a lower accumulation value of 3.59 mg plant^−1^ than the other treatments (Table S4).

Figure 4c,d shows that differences among treatments were more pronounced for shoot nitrogen accumulation than for shoot nitrogen concentration, with the greatest differences occurring under W2 and W3.

In narrative terms, calculated nitrogen accumulation was closely tied to total biomass dynamics. Both root and shoot nutrient pools decreased concurrently with stress duration, with the biostimulant maintaining higher descriptive values throughout.

## 3. Discussion

### 3.1. Effects of Seed Treatment on Germination and Initial Vigor

This study aimed to evaluate the duration of the biological effects of a seed-applied biostimulant following delayed watering after sowing in dry soil. Under autumn conditions, wheat seeds may remain in the soil for extended periods without initiating germination because of insufficient moisture. Prolonged exposure to dry conditions may subsequently affect seedling vigor through physiological deterioration processes associated with seed ageing [3,4].

Under these conditions, the final germination percentage was not influenced by the application of either the fungicide or the biostimulant and remained high in all treatments. However, biostimulant application significantly improved germination performance, as indicated by reductions in mean germination time (MGT) of up to 9.4 h for BS and 8.7 h for TBS, irrespective of the watering regime. While a reduction of several hours represents a statistically significant acceleration of the germination process under controlled conditions, its agronomic relevance within a real-world production system should be interpreted with caution. In absolute terms, reductions of 8.7–9.4 h constitute a relatively minor fraction of the extended timeframe required for emergence under prolonged water deficit. Nonetheless, on a physiological level, a shorter MGT is considered an indicator of higher seed vigor and a greater capacity for crop establishment and is associated with faster seedling development during the early growth stages [2,3]. The reduction in MGT therefore indicates a faster germination response following moisture restoration, rather than demonstrating improved agronomic performance or drought tolerance.

The high final germination percentage across treatments also indicates that the seed lot had high initial germination potential. The effects of seed treatment became more apparent when watering was delayed, particularly after two to three weeks, when the combined treatment showed a stronger biological response than the untreated control. The higher numerical values observed for the combined treatment compared with the fungicide treatment alone suggest a possible mitigating effect of the biostimulant on the lower growth response associated with triticonazole treatment [11,30]. Because a formal fungicide × biostimulant interaction was not separately evaluated, this observation should be interpreted as a treatment-associated trend rather than as evidence of a statistically demonstrated compensatory interaction.

### 3.2. Root Architecture and Biomass

Following radicle emergence, the active growth phase begins, and radicle and coleoptile elongation become dependent on the efficiency of reserve mobilization and the metabolic activity of the embryo [31]. In this context, application of the biostimulant Oligal SD accelerated initial growth, as reflected by greater total seedling length in Petri dishes. This response suggests a positive effect on early vigor and crop establishment and is consistent with the findings reported by Finch-Savage and Bassel [2].

By contrast, treatment with the triticonazole-based fungicide alone had an inhibitory effect on radicle growth, particularly when watering was delayed for four to five weeks, when the effects of prolonged dry exposure became more evident. Although triticonazole provides protection against soil-borne pathogens [32], its inhibitory effect on root elongation may also be related, at least partly, to its influence on hormonal metabolism, particularly gibberellin biosynthesis, which may reduce cell elongation in roots [9,10].

Nevertheless, the greatest growth values were recorded for the combined treatment, suggesting that the biostimulant may have contributed to maintaining greater initial growth in the presence of triticonazole. This response may reflect greater initial seed vigor and a more effective resumption of growth after moisture restoration. Biostimulant-associated effects on oxidative balance have also been proposed in previous studies [14,15,19,33], which is compatible with the stronger response observed for the combined treatment.

The combined treatment also promoted the development of additional seminal roots and resulted in greater root biomass at BBCH 11–12. This response may be related to the bioactive compounds and micronutrients supplied by the biostimulant, including boron, which is involved in cell division and meristematic tissue development [18,20]. The greater root development observed at these early stages may favor subsequent resource acquisition after rehydration; however, the present study measured early root traits rather than direct water or nutrient uptake.

Root biomass was analyzed BBCH 11 (first true leaf unfolded) and BBCH 12 (two true leaves fully unfolded) to evaluate treatment-associated differences during early plant development. The greater root biomass recorded when the biostimulant was applied in combination with triticonazole was consistent with the greater seminal root development observed in the treated variants [34]. In addition, a contribution from the micronutrients contained in the biostimulant, particularly boron, is plausible considering its role in maintaining cell-wall integrity, tissue elasticity, and the functioning of apical meristems [35]. However, because our measurements were strictly confined to these very early developmental stages (BBCH 11 and BBCH 12), it cannot be assumed that these initial differences in root biomass will necessarily persist throughout the later vegetative and reproductive stages of the crop. Thus, the observed response should be interpreted as enhanced early root development rather than evidence of sustained improvement in root-system architecture or drought tolerance.

### 3.3. Nitrogen Nutrition and Shoot Growth

In addition to its effects on root-system development, the biostimulant significantly influenced nitrogen accumulation and dynamics within the plants [16,17]. Root nitrogen concentrations of 2.0–2.5% total N were within the physiological range reported for wheat during the early growth stages at BBCH 12 [36]. The higher values observed in the biostimulant-treated variants may indicate more effective nitrogen acquisition during early establishment, consistent with their greater seminal root development and root biomass [16]. The higher calculated nitrogen accumulation in treated variants was associated with greater root biomass and therefore cannot be attributed solely to differences in tissue nitrogen concentration. This response may reflect a greater capacity for nitrogen acquisition following rehydration and utilization of available nitrogen resources [26,37,38]. However, because nitrogen-assimilation pathways and enzyme activities were not directly assessed, the contribution of specific nitrogen metabolic processes or changes in nitrogen-use efficiency cannot be confirmed from the present results.

A high nitrogen content in roots is associated with greater nitrogen transport to the shoots [27]. The nitrogen concentrations determined in the shoots indicated adequate nitrogen nutrition during the early growth stages, while calculated plant nitrogen accumulation was generally higher in the biostimulant-treated variants. The greater shoot N status was accompanied by greater first leaf length, shoot height, and shoot dry weight, indicating that the response to the biostimulant extended beyond tissue N concentration to early biomass formation. The significantly greater first leaf length and shoot height recorded in TBS may be associated with the bioactive compounds in the biostimulant and with the presence of manganese, which is involved in redox processes and may support nitrogen assimilation [39,40]. However, these relationships should be interpreted as associations rather than evidence of a direct stimulation of nitrogen metabolism.

Overall, the higher N accumulation in biostimulant-treated plants, together with greater root biomass and shoot growth, suggests a more favorable nitrogen status during early establishment. Because measurements were restricted to BBCH 11–12, these responses indicate improved early nutritional and growth status rather than demonstrated improvements in later nitrogen-use efficiency or crop productivity.

### 3.4. Stress Factor: Delayed Watering for Two to Five Weeks

The positive effects of biostimulants under abiotic stress conditions have been widely reported [41,42,43]. In the present study, the biological response associated with the biostimulant was strongest after two to three weeks of delayed watering, remained detectable after approximately four weeks, and became substantially attenuated after five weeks. This pattern indicates that the magnitude of the biological response varied with the duration of the pre-germination dry period, with the strongest response occurring within the two- to three-week interval under the experimental conditions. Although seed viability may be maintained, the progressive loss of vigor associated with prolonged dry exposure may limit the capacity of seeds to rapidly resume germination and growth after favorable moisture conditions are restored [3,4,7].

The stronger response after two to three weeks may be related to the capacity of physiologically competent seeds to resume metabolic activity after rehydration. Seed imbibition initiates membrane repair, reserve mobilization, and renewed metabolic activity, whereas prolonged dry storage can progressively impair these processes through seed ageing [3,4,7]. In this context, the bioactive compounds supplied by the biostimulant may have supported post-rehydration recovery, potentially through processes associated with osmotic adjustment and redox regulation [19,44].

Recent studies in wheat have likewise shown that seed-applied or microbial biostimulants can modify plant responses to water deficit, although the magnitude and nature of these responses vary with the biostimulant, genotype, and stress conditions [12,45]. The substantial reduction in treatment-associated growth after five weeks may, in turn, reflect increasing physiological constraints imposed by prolonged dry exposure. Seed ageing is associated with oxidative imbalance and deterioration of membrane integrity, which can progressively compromise cellular repair and metabolic recovery after rehydration [3,4,7]. Thus, as the dry period extends, the physiological limitations of the seed may increasingly restrict the expression of treatment-associated benefits. A complementary hormonal mechanism may also be considered, as ABA and GA are major and antagonistic regulators of seed dormancy and germination in cereals [46]. A shift toward a less favorable ABA/GA balance under prolonged stress could therefore contribute to slower post-imbibition growth. Taken together, these processes provide plausible physiological explanations for the temporal pattern observed in the present study, although oxidative damage, membrane integrity, and ABA/GA dynamics were not directly assessed.

The stronger response of the combined fungicide and biostimulant treatment after two to three weeks, followed by a reduced advantage after four to five weeks, is consistent with a treatment-associated biological response that was most evident while seed physiological status remained sufficiently preserved. The inhibitory effect of triticonazole on growth was reflected by values lower than those recorded in the untreated control [10]. However, because a formal fungicide × biostimulant interaction was not separately tested, the higher values observed for the combined treatment should not be interpreted as evidence of a statistically demonstrated compensatory interaction. Rather, they indicate that the biological response associated with the biostimulant remained detectable in the presence of the fungicide under some watering regimes.

The reduction in growth observed when watering was delayed by four or five weeks may also have been influenced by the climatic conditions prevailing during emergence. Plants supplied with water after four weeks developed during October, when temperatures were lower, cloud cover was greater, and day length was shorter. These conditions may have represented an additional factor contributing to the reduction in growth rate. For this reason, the differences observed among watering regimes should be interpreted cautiously, because the respective treatments were established at different times and therefore experienced different environmental conditions.

The greater seminal root development, root biomass, and nitrogen accumulation observed in treated plants provide additional support for a more favorable early recovery following rehydration. However, these responses should be interpreted as indicators of improved early growth and nutritional status rather than as evidence of enhanced drought tolerance or directly measured increases in water or nutrient uptake.

Overall, the results indicate that the magnitude of the biological response associated with the seed-applied biostimulant depended on the duration of the pre-germination dry period. The strongest response occurred after two to three weeks, remained detectable after approximately four weeks, and declined substantially after five weeks. Importantly, this decline should not be interpreted as evidence of chemical degradation or loss of product activity, because the chemical persistence or stability of the biostimulant was not independently assessed. Similarly, seed physiological status was not characterized before and after the dry period; therefore, the present study cannot determine whether the reduced response after five weeks resulted primarily from progressive seed deterioration, reduced treatment-associated activity, or both.

Although keeping seeds in a dry substrate for two to five weeks provides a controlled model of delayed rainfall after sowing, actual field conditions are considerably more complex. Factors such as dynamic soil temperature, relative humidity, soil texture, matric potential, microbial activity, seed-to-soil contact, oxygen availability, and intermittent moisture events can influence both seed physiological status and treatment-associated responses [47].

## 4. Materials and Methods

### 4.1. Experimental Site

The experiments were conducted under controlled conditions at the Plant Nutrition and Soil Fertility Laboratory of the “Ion Ionescu de la Brad” Iași University of Life Sciences, Romania.

The study aimed to evaluate the effect of seed treatment with the Oligal SD biostimulant (Timac Agro, Bucharest, Romania) on germination capacity and subsequent plant vigor under conditions of water deficit at sowing and a prolonged absence of precipitation, which are characteristic of regions with a strongly continental climate. For this purpose, the treated seeds were maintained under moisture-deficit conditions for up to five weeks before watering.

This approach reproduces water-stress conditions frequently encountered in early autumn and allows the performance of treated seeds to be evaluated until favorable moisture conditions are established.

### 4.2. Experimental Design

The study comprised four seed treatments evaluated under five distinct post-sowing watering regimes to determine the effect of seed treatment with the Oligal SD biostimulant, applied either alone or in combination with Premis (BASF, Ludwigshafen, Germany) fungicide, on seed performance after different periods of delayed watering. Treatment effects were evaluated separately within each watering regime.

Two independent experiments were established using different growth systems designed to complement each other: (i) Petri dishes for evaluating germination under controlled laboratory conditions and (ii) growth pots for evaluating emergence and early growth in a substrate that reproduced field conditions at an experimental scale.

The seed treatments and watering regimes were identical in both experiments:

1. Seed treatment, with four levels: untreated control (C), treatment with a triticonazole-based fungicide (T), treatment with the Oligal SD biostimulant (BS), and combined fungicide + biostimulant treatment (TBS).
2. Watering regime, defined by the timing of the first watering after sowing, with five levels: watering at sowing (W1), watering after two weeks (W2), watering after three weeks (W3), watering after four weeks (W4), and watering after five weeks (W5).

For each watering regime, each of the four seed treatments was established in three independent replicates. In the Petri-dish experiment, 20 treatment-by-watering combinations were evaluated, each represented by three independent Petri dishes, resulting in a total of 60 experimental units.

In the growth-pot experiment, separate sets of pots were used for destructive measurements at BBCH 11 and BBCH 12. Thus, 40 treatment-by-watering-by-growth-stage combinations were evaluated, each represented by three independent pots, resulting in a total of 120 pots. The BBCH 11 and BBCH 12 stages represented destructive sampling times and were not considered experimental factors.

In the control treatment, seeds were treated with 5 mL of bidistilled water kg^−1^. The fungicide treatment consisted of the application of 1.5 mL of Premis 25% SC and 3.5 mL of bidistilled water kg^−1^, whereas the biostimulant treatment consisted of 3 mL of Oligal SD and 2 mL of bidistilled water kg^−1^. In the combined treatment (TBS), the seeds were first treated with the fungicide and allowed to dry. After 8 h, the biostimulant was applied under the same conditions as in the BS treatment. To ensure that all treatments received the same total application volume (10 mL kg^−1^ of seed), the C, T, and BS treatments received an additional application of 5 mL kg^−1^ of bidistilled water at the corresponding application time. The application rates were those recommended by the manufacturer, and the addition of bidistilled water ensured uniform distribution of the solution over the seed surface.

Oligal SD contains 1.5% Zn, 1.5% Mn, 0.01% B, and the Fertiactyl complex, based on humic and fulvic acids and bioactive compounds. The fungicide used was Premis 25% SC, a triticonazole-based product with contact and systemic activity.

The biological material consisted of winter wheat seeds (*Triticum aestivum* L., cv. Glosa) purchased from an authorized supplier. Seed treatment was performed in the laboratory. The treatments were applied manually by spraying 5 mL of solution kg^−1^ of seeds, followed by continuous mixing and drying at room temperature until the following day.

The watering regimes were designed to reproduce the hydroclimatic conditions characteristic of the Moldavian Plateau in northeastern Romania, based on meteorological data recorded by the Romanian National Meteorological Administration between 2010 and 2024. Analysis of these data indicates substantial variability in autumn precipitation, characterized by alternating dry years and years with normal precipitation. Under these conditions, sowing may frequently take place in soil with insufficient moisture, and crop emergence subsequently depends on the occurrence of rainfall. Recent studies have also shown that drought frequently affects winter wheat crops in this region, highlighting the need for new approaches to help plants cope with water shortage [48].

The watering amounts were converted into equivalent volumes corresponding to the surface area of each pot. Distilled water was used to avoid the influence of salinity or other dissolved compounds on germination. Water was applied manually according to the procedures described below for each experimental system and set of growing conditions.

### 4.3. Experimental Setup, Growth Conditions, and Measurements

The Petri-dish experiment was designed to evaluate the initial physiological response of the seeds under strictly controlled conditions, whereas the growth-pot experiment allowed plant development to be assessed in a substrate under controlled conditions simulating a soil-based growth environment at an experimental scale. The results obtained in the two systems are presented in parallel to highlight the consistency and continuity of the treatment effects on germination and early growth following delayed watering.

#### 4.3.1. Petri-Dish Experiment

##### Preparation of the Petri Dishes

For each experimental unit, 50 seeds treated according to the experimental design were counted and placed between sheets of germination paper using the between-paper method (BP), in accordance with standard seed-germination testing procedures. Three replicates were established for each factorial combination.

Moistening was performed at the times specified by the experimental factors by spraying the germination paper with a controlled volume of distilled water. The volume was sufficient to ensure complete hydration of the germination paper without producing excess free water. The moisture content of the germination paper was monitored daily and maintained by adding distilled water when necessary to prevent substrate dehydration. The dishes were maintained at a constant temperature of 20 ± 0.5 °C, and germination took place in darkness in accordance with ISTA recommendations [49].

##### Biometric Measurements

Biometric measurements were performed in accordance with ISTA protocols and the Seedling Evaluation Handbook, following standardized germination-testing procedures with specific adaptations to the experimental conditions [49].

Measurements were performed 72 h after moistening the germination paper on a sample of 10 seedlings per dish. The seedlings were selected from seeds that had germinated on the second day, when the germination percentage exceeded 80%. Seeds were considered germinated when the radicle had reached a length of at least 1 mm.

The seedlings included in the analysis were selected based on morphological uniformity to ensure the representativeness of each experimental unit. Measurements were performed at the same time for all treatments to avoid the influence of differences in germination speed.

Growth stages were defined according to the BBCH scale [50]. For the Petri-dish experiment, BBCH 09 was characterized under laboratory conditions by the appearance and elongation of the coleoptile, while in the growth-pot experiment, BBCH 11 and BBCH 12 corresponded to the first and second true leaves unfolded, respectively.

The following parameters were evaluated: germination percentage (G%), mean germination time (MGT, h), total seminal root length (TSRL, mm), coleoptile length (CL, mm), seedling fresh weight (FW, mg seedling^−1^), seedling dry weight (DW, mg seedling^−1^), and seed vigor index (SVI).

Germination percentage was determined on the seventh day by counting the germinated seeds in each dish. G% was calculated by dividing the number of germinated seeds by the 50 seeds used per dish and expressing the result as a percentage.

Mean germination time was determined by evaluating the seeds at 12 h intervals after moistening, and the results were expressed in hours. MGT was calculated according to Ellis and Roberts [51] and Ranal and Santana [52]:

MGT = Σ(n × t)/Σn,(1)

where (n) represents the number of seeds germinated at time (t), (t) is the time elapsed from the beginning of the germination test (expressed in hours), and (Σn) represents the total number of seeds germinated by the end of the experiment. Germination counts were performed at 12-h intervals.

Coleoptile length (CL), primary root length (PRL), and total seminal root length (TSRL) were determined using millimeter-grid paper. TSRL was calculated as the sum of the lengths (mm) of the primary seminal root and all other seminal roots of each seedling [53].

The seed vigor index was calculated according to the standard method described by Abdul-Baki and Anderson [54], using the following equation:

SVI = G(%) × (CL + PRL),(2)

where G% represents germination percentage, CL represents coleoptile length in millimeters, and PRL represents primary root length in millimeters.

#### 4.3.2. Growth-Pot Experiment

##### Preparation of the Pots and Substrate

The metal growth pots had an internal diameter of 20 cm and a height of 25 cm. Drainage holes were provided at the base, and each pot was placed individually on a container used to collect percolated water.

The substrate consisted of a mixture containing 30% peat, 30% well-decomposed manure, 30% black soil, and 10% sand. The mixture was homogenized, air-dried, cleared of plant residues, and passed through a 2 mm sieve to obtain uniform material.

The pots were filled manually by gradually adding the substrate prepared in the laboratory. The substrate was uniformly compacted to ensure a constant bulk density. Approximately 7 kg of mixture was added to each pot, producing a substrate column approximately 24 cm high. The substrate moisture content was adjusted to approximately 30% of field capacity at the time of pot filling. Field capacity was determined gravimetrically, and the corresponding initial substrate moisture content was 13.6% (*w*/*w*). No additional water was supplied between pot filling and sowing. The preparation and filling procedures were identical for all experimental treatments.

The main chemical characteristics of the substrate components are presented in Table 1.

##### Wheat Growing Conditions in Pots

Twenty-five seeds were sown in each pot and covered with a 2 cm layer of substrate. Sowing was performed four days after preparation and filling of the pots to allow the substrate to settle. All pots were sown on the same day. No additional water was supplied from pot preparation until the scheduled first watering for each treatment, allowing the substrate moisture to decline progressively during the pre-germination period.

The five watering regimes (W1–W5) represented different durations of delayed first watering. The W1 pots received water immediately after sowing, whereas the W2, W3, W4, and W5 pots received their first watering after 14, 21, 28, and 35 days, respectively. Thus, W1–W5 represented different durations of pre-germination dry exposure rather than constant soil-moisture levels. No emergence was observed in the non-watered pots before their scheduled first watering.

After emergence, the seedlings were thinned to leave 20 uniformly developed plants in each pot. The final density was equivalent to approximately 500 plants m^−2^ to simulate field conditions in terms of the space available for plant nutrition. Each treatment was established in three replicates, with each pot representing a separate experimental unit.

The growth pots were maintained in the laboratory greenhouse under natural conditions without artificial control of temperature or lighting. Mean temperatures were characteristic of the period evaluated, September–November 2024, in the study area. The watering regime was the only factor that was strictly controlled and was applied according to the experimental protocol.

Water was applied manually and slowly to ensure uniform infiltration into the substrate and to avoid surface runoff. At the first watering, the substrate in each pot was brought to gravimetrically determined field capacity. Thereafter, substrate moisture was monitored daily and maintained close to field capacity by adding the required volume of distilled water. Because substrate moisture was not quantitatively monitored during the pre-watering period, the W1–W5 treatments were defined by the duration of delayed first watering rather than by precisely quantified soil-moisture levels.

##### Biometric Measurements of Wheat Plants

The following parameters were determined in the growth-pot experiment: emergence rate (ER%), seminal root number (SR), shoot height (SH), first leaf length (FLL), and root and shoot dry weight at BBCH 11 and BBCH 12.

Emergence rate was determined by counting the emerged plants in each pot and expressing the result as a percentage according to the following equation:

ER (%) = (EP/SS)×100,(3)

where EP represents the number of emerged plants, including those subsequently removed during thinning, and SS represents the number of seeds sown in each pot, namely 25 seeds.

Morphological measurements were performed on a sample of 20 plants per replicate. The plants were carefully removed from the pots, and the roots were washed under a low-pressure stream of water to remove the substrate. The plants were subsequently placed individually on trays and measured using a ruler graduated in millimeters.

First leaf length was measured from the point of insertion on the stem to the tip of the leaf blade. Shoot height was determined by measuring the maximum extended leaf length from the crown to the tip of the longest leaf blade.

Seminal root number was determined by visually counting the roots originating from the scutellar node after the substrate had been completely removed. Values were recorded separately for each plant.

Root dry weight (RDW) and shoot dry weight (SDW) were determined for the entire sample of plants because separating the roots of individual plants was difficult. The roots were cut at the level of the caryopsis and oven-dried at 70 °C for 48 h using a laboratory oven (POL-EKO-APARATURA, Wodzisław Śląski, Poland) until constant weight, which was verified by two consecutive weighings on an analytical balance (Kern & Sohn GmbH, Balingen, Germany). The results were expressed as mg plant^−1^ by dividing the total dry weight by the number of plants analyzed (20). The same procedure was used to determine shoot dry weight. Measurements were performed at the BBCH 11 and BBCH 12 growth stages.

##### Determination of Nitrogen Concentration and Accumulation

To evaluate the nutritional status of the wheat plants, the total nitrogen concentration (Nt conc.) was determined using the micro-Kjeldahl method. The chemical analysis was performed on the entire plant sample from each replicate, processing the shoots and roots separately for each experimental unit. The collected plant tissues were dried, finely ground, and subjected to sulfuric acid digestion block (Gerhardt, Königswinter, Germany), followed by distillation using an automated distillation unit (Velp Scientifica, Usmate Velate, Italy) and titration. The final plant nitrogen concentration was expressed as a percentage (%) of dry matter. Based on the dry biomass (DW) and the determined chemical concentration, the total nitrogen accumulation (Nt acc.) in the plant was calculated as an indicator of absolute nutrient uptake capacity. The nitrogen accumulation was determined separately for the shoots and roots, using the following formula:

Nacc (mg plant^−1^) = Nt conc. (%) × DW (mg plant^−1^)/100 (4)

This approach allowed for the evaluation of both the nutritional status of the tissues and the absolute amount of nitrogen absorbed by the wheat plants under the influence of the experimental treatments.

##### Substrate Analysis Methods

The chemical and physical analyses of the substrate were performed using standard pedological and agrochemical methods recommended by the Romanian National Research and Development Institute for Soil Science, Agrochemistry and Environmental Protection, in accordance with the specialized Romanian methodological literature and supplemented with internationally recognized procedures [55].

Soil reaction (pH) was determined potentiometrically in a 1:2.5 soil-to-water suspension using a digital pH meter (Hanna Instruments, Woonsocket, RI, USA), according to standard procedures for evaluating soil reaction.

Total nitrogen (Nt) was determined using the Kjeldahl method. Mineral nitrogen (NO_3_^−^ + NH_4_^+^) was determined by extraction with KCl solution followed by distillation with Devarda’s alloy using an automated distillation unit (VELP Scientifica, Usmate, Italy). Available phosphorus (P_2_O_5_) and potassium (K_2_O) were determined using the Egner–Riehm–Domingo method with ammonium lactate–acetate solution as the extractant. Extractable phosphorus (P-AL) was quantified spectrophotometrically using a UV-1700 spectrophotometer (Shimadzu Corp., Kyoto, Japan), whereas extractable potassium (K-AL) was determined by flame photometry using a Flame Photometer 410 (Sherwood Scientific Ltd., Cambridge, UK). [56].

### 4.4. Statistical Analysis

Statistical analyses were performed separately for each watering regime (W1–W5). Within each watering regime, the effect of seed treatment (C, T, BS, and TBS) was evaluated by one-way analysis of variance (ANOVA). When significant treatment effects were detected, mean separation was performed using Tukey’s honestly significant difference (HSD) post hoc test at a significance threshold of *p* ≤ 0.05.

The three replicates represented independent experimental units and were not included as an experimental factor in the statistical model. For the Petri-dish experiment, each Petri dish represented one replicate. For the growth-pot experiment, each pot represented one replicate, and data obtained at BBCH 11 and BBCH 12 were analyzed separately because destructive sampling was performed at each growth stage.

No statistical comparisons were performed among watering regimes, and no treatment × watering interaction was tested. Accordingly, the letters indicating significant differences in tables and figures refer exclusively to comparisons among seed treatments within the same watering regime and should not be compared across W1–W5.

All statistical analyses were performed using SPSS version 22 (IBM Corp., Armonk, NY, USA).

## 5. Conclusions

The seed-applied biostimulant provided measurable biological benefits after treated seeds were maintained in dry soil conditions and support early seedling development under pre-germinative water-stress conditions. Our results indicate that biological effects associated with the seed-applied biostimulant remained detectable following delayed watering, as reflected by reduced mean germination time (MGT) and greater seminal root development in growth pots, with the strongest response observed when watering was delayed by 2–3 weeks. Under the conditions of this experiment, the results demonstrate that, although the biostimulant supports enhanced early seminal root development after moisture restoration, these effects reflect localized, early-stage responses within the growth pots (up to BBCH 12) rather than broader, unquantified improvements to the overall mature root system architecture.

The magnitude of these treatment effects depended on the duration of the dry period and is likely modulated by compounding soil and environmental factors. The biostimulant treatment was also associated with greater root elongation in the presence of the triticonazole-based fungicide, particularly after delayed watering of up to four weeks.

These benefits progressively decreased toward the fifth week, indicating that the magnitude of the treatment response became less pronounced as the pre-germination dry period was extended. Because the underlying processes related to nitrogen metabolism and stress responses were not assessed directly, and because seed physiological status and biostimulant persistence were not independently evaluated during the dry period, the mechanisms underlying this attenuation cannot be determined unequivocally from the present experiment.

Therefore, the two- to three-week period identified here should be regarded as the interval associated with the strongest biological response under the experimental conditions, rather than as a universal limit of treatment effectiveness. Field validation under variable soil and environmental conditions is required before these findings can be translated into recommendations for autumn sowing in drought-prone regions.

## Figures and Tables

**Table 1:** Experimental substrate composition.

Soil Mixture	% in Substrate	pH (H_2_O)	Nt (%)	NO_3_ + NH_4_ (ppm)	P-AL (%)	K-AL (%)
Peat	30	6.3	0.12	8	0.005	0.016
Composted manure	30	6.8	0.92	100	0.280	0.800
Topsoil	30	7.5	0.28	60	0.006	0.038
Sand	10	6.3	0.03	0.2	0.001	0.006

pH, potential of hydrogen; Nt, total nitrogen; NO_3_ + NH_4_, mineral nitrogen; P-AL, mobile phosphorus; K-AL, mobile potassium.

## Data Availability

The original contributions presented in this study are included in the article/Supplementary Material. Further inquiries can be directed to the corresponding author.

## Supplementary Materials

The following supporting information can be downloaded at https://www.mdpi.com/article/10.3390/plants15172552/s1, Table S1: Germination parameters and seedling vigor of wheat seeds grown in Petri dishes (BBCH 07–09) as affected by seed treatment and watering regime; Table S2: Effects of seed treatment and watering regime on morphological parameters of wheat seedlings at BBCH 12; Table S3: Root and shoot dry weight of wheat seedlings at BBCH 11–12 under different seed treatments and watering regimes; Table S4: Nitrogen concentration and accumulation in roots and shoots of wheat seedlings at BBCH 12 under different seed treatments and watering regimes.
