# Supplementary material for: Temporal Persistence of the Biological Effects of a Seed-Applied Biostimulant on Wheat (Triticum aestivum) Germination and Early Growth Under Extended Pre-Germination Soil Drought
Source: Plants (Basel). 2026 Aug 22;15(17):2552. doi: 10.3390/plants15172552 (PMC13567095; doi:10.3390/plants15172552)
Supplement: Supplementary file 1 [file plants-15-02552-s001.zip › plants-4472992-supplementary.pdf]

Table S1. Germination parameters and seedling vigor of wheat (*Triticum aestivum*) seeds grown in Petri dishes (BBCH 07–09) as affected by seed treatment and watering regime.

Watering	Trt.	Germination rate (%)	GMT (houres)	SVI	*TSRL (mm)	CL (mm)
**W1**	**C**	94.0 ± 0.58 a	55.5 ± 0.38 a	1481 ± 86.1 b	25.0 ± 1.53 b	4.2 ± 0.44 b
	**T**	94.3 ± 1.45 a	52.5 ± 0.18 b	1433 ± 63.3 b	22.3 ± 1.67 b	4.7 ± 0.17 b
	**BS**	95.7 ± 0.33 a	48.2 ± 0.22 c	2332 ± 40.6 a	42.7 ± 1.45 a	7.0 ± 0.29 a
	**TBS**	96.3 ± 0.33 a	47.7 ± 0.29 c	2572 ± 60.5 a	50.0 ± 2.08 a	6.8 ± 0.17 a
**W2**	**C**	94.7 ± 0.67 a	55.5 ± 0.75 a	1379 ± 69.9 b	24.7 ± 1.46 c	3.3 ± 0.33 b
	**T**	95.3 ± 0.67 a	53.2 ± 0.23 a	1390 ± 75.0 b	22.7 ± 1.08 c	4.0 ± 0.58 b
	**BS**	96.3 ± 0.88 a	46.1 ± 0.64 b	2341 ± 41.6 a	38.9 ± 1.68 b	7.7 ± 0.33 a
	**TBS**	95.7 ± 0.67 a	46.8 ± 0.60 b	2572 ± 68.4 a	46.5 ± 1.53 a	8.0 ± 0.58 a
**W3**	**C**	95.7 ± 1.20 a	54.5 ± 0.47 a	1469 ± 42.2 b	25.0 ± 2.21 b	4.0 ± 0.50 b
	**T**	96.0 ± 0.58 a	51.2 ± 0.38 b	1377 ± 20.5 b	21.5 ± 0.50 b	4.2 ± 0.17 b
	**BS**	96.7 ± 0.88 a	46.9 ± 0.80 c	2345 ± 50.1 a	39.3 ± 1.16 a	8.2 ± 0.44 a
	**TBS**	96.7 ± 0.33 a	47.4 ± 0.38 c	2553 ± 83.8 a	44.7 ± 1.99 a	8.5 ± 0.29 a
**W4**	**C**	95.7 ± 0.88 a	55.9 ± 0.22 a	1432 ± 16.9 c	24.4 ± 0.34 b	3.8 ± 0.17 c
	**T**	96.3 ± 0.88 a	53.5 ± 0.47 b	1296 ± 27.9 c	18.9 ± 0.20 c	4.2 ± 0.17 c
	**BS**	96.3 ± 1.20 a	47.3 ± 0.29 c	2274 ± 41.8 b	41.8 ± 1.43 a	6.7 ± 0.33 b
	**TBS**	97.0 ± 0.58 a	47.6 ± 0.21 c	2587 ± 97.5 a	46.0 ± 1.77 a	8.3 ± 0.33 a
**W5**	**C**	94.0 ± 0.58 a	58.7 ± 0.27 a	1280 ± 35.3 b	22.0 ± 1.04 b	3.5 ± 0.29 b
	**T**	94.7 ± 0.88 a	57.2 ± 0.17 a	1125 ± 87.1 b	18.5 ± 0.35 c	3.8 ± 0.44 b
	**BS**	95.7 ± 1.20 a	49.2 ± 0.37 c	2028 ± 137.7 a	37.7 ± 0.71 a	6.8 ± 0.44 a
	**TBS**	95.3 ± 1.20 a	50.8 ± 0.58 b	2399 ± 171.6 a	40.7 ± 0.33 a	8.2 ± 0.44 a

Trt. – treatment; C – Control; T – Triticonazole; BS – Biostimulant; TBS – Triticonazole + Biostimulant. GMT – Germination Mean Time; TSRL – Total Seminal Root Length; CL – Coleoptile Length; SVI – Seed Vigor Index. W1–W5 – watering intervals (W1: immediate; W2–W5: watering at 2–5 weeks after sowing). Data are presented as mean ± SE ($n = 3$). Different letters indicate statistically significant differences among seed treatments within each watering regime according to Tukey's test ($p < 0.05$). Letters should not be compared across watering regimes.

Table S2. Effects of seed treatment and watering regime on morphological parameters of wheat (Triticum aestivum) seedlings at BBCH 12.

**Watering**	Trt.	FLL	SH	SR
**W1**	**C**	16.4 ± 0.74 b	22.8 ± 0.72 c	3.5 ± 0.14 b
	**T**	18.4 ± 0.52 ab	26.1 ± 0.82 b	4.1 ± 0.17 b
	**BS**	18.5 ± 0.43 ab	25.5 ± 0.74 b	3.9 ± 0.17 b
	**TBS**	20.4 ± 0.50 a	28.9 ± 0.51 a	4.9 ± 0.20 a
**W2**	**C**	16.3 ± 0.78 b	22.2 ± 0.87 b	3.4 ± 0.13 c
	**T**	17.3 ± 1.26 ab	26.5 ± 1.03 a	4.1 ± 0.22 b
	**BS**	18.2 ± 0.42 ab	25.7 ± 0.75 a	3.7 ± 0.13 bc
	**TBS**	20.2 ± 0.49 a	28.6 ± 0.55 a	5.0 ± 0.21 a
**W3**	**C**	16.1 ± 0.60 c	23.8 ± 0.78 b	3.4 ± 0.13 b
	**T**	17.1 ± 0.44 bc	26.4 ± 0.56 ab	4.0 ± 0.20 b
	**BS**	19.1 ± 0.83 b	26.7 ± 0.91 a	3.9 ± 0.21 b
	**TBS**	21.7 ± 0.65 a	26.7 ± 0.69 a	4.9 ± 0.20 a
**W4**	**C**	13.8 ± 0.51 b	23.0 ± 0.48 b	3.5 ± 0.17 b
	**T**	14.0 ± 0.58 b	22.4 ± 0.69 b	3.8 ± 0.17 b
	**BS**	15.7 ± 0.68 b	23.7 ± 0.68 b	4.0 ± 0.17 b
	**TBS**	17.5 ± 0.42 a	27.0 ± 0.41 a	4.7 ± 0.20 a
**W5**	**C**	11.4 ± 0.34 a	18.5 ± 0.41 c	3.5 ± 0.14 b
	**T**	10.7 ± 0.32 a	17.7 ± 0.47 c	3.6 ± 0.17 ab
	**BS**	11.2 ± 0.33 a	21.9 ± 0.26 a	4.0 ± 0.17 ab
	**TBS**	10.5 ± 0.36 a	20.0 ± 0.27 b	4.1 ± 0.18 a

Trt. – treatment; C – Control; T – Triticonazole; BS – Biostimulant; TBS – Triticonazole + Biostimulant. FLL – First Leaf Length; SH – Shoot Height; SR – Seminal Roots. W1–W5 – watering intervals (W1: immediate; W2–W5: watering at 2–5 weeks after sowing). Data are presented as mean ± SE (n = 3). Different letters indicate statistically significant differences among seed treatments within each watering regime according to Tukey's test ($p < 0.05$). Letters should not be compared across watering regimes.

Table S3. Root and shoot dry weight of wheat (Triticum aestivum) seedlings at BBCH 11–12 under different seed treatments and watering regimes.

		BBCH 11		BBCH 12	
**Watering**	**Trt.**	**RDW (mg plant⁻¹)**	**SDW (mg plant⁻¹)**	**RDW (mg plant⁻¹)**	**SDW (mg plant⁻¹)**
**W1**	**C**	11.1 ± 0.4 b	17.5 ± 0.2 b	47.0 ± 0.6 d	132.4 ± 1.0 c
	**T**	12.3 ± 0.3 ab	19.1 ± 0.1 a	57.4 ± 0.4 c	168.4 ± 1.8 b
	**BS**	12.7 ± 0.2 a	18.9 ± 0.1 a	65.0 ± 0.3 b	182.3 ± 1.4 a
	**TBS**	12.8 ± 0.2 a	18.6 ± 0.2 a	71.5 ± 0.5 a	188.8 ± 1.7 a
**W2**	**C**	12.4 ± 0.3 b	18.7 ± 0.2 b	51.3 ± 0.5 d	139.4 ± 0.5 d
	**T**	12.6 ± 0.3 ab	19.6 ± 0.2 a	59.1 ± 1.1 c	152.5 ± 2.2 c
	**BS**	12.9 ± 0.3 ab	19.6 ± 0.1 a	77.1 ± 0.4 b	210.7 ± 2.9 b
	**TBS**	13.8 ± 0.2 a	20.1 ± 0.2 a	84.5 ± 0.3 a	229.7 ± 2.5 a
**W3**	**C**	12.5 ± 0.3 b	19.5 ± 0.3 c	53.3 ± 0.5 c	156.1 ± 2.1 b
	**T**	13.4 ± 0.4 ab	20.3 ± 0.1 b	58.7 ± 0.6 b	162.3 ± 1.0 b
	**BS**	12.6 ± 0.3 b	19.2 ± 0.1 c	69.9 ± 1.1 a	206.7 ± 2.6 a
	**TBS**	13.9 ± 0.2 a	21.2 ± 0.2 a	73.1 ± 1.3 a	214.8 ± 2.2 a
**W4**	**C**	12.0 ± 0.4 a	18.4 ± 0.1 b	37.4 ± 0.3 c	112.7 ± 4.4 b
	**T**	12.5 ± 0.1 a	18.9 ± 0.2 b	36.5 ± 0.3 c	111.7 ± 4.4 b
	**BS**	12.1 ± 0.2 a	18.7 ± 0.1 b	45.7 ± 0.7 b	147.9 ± 2.7 a
	**TBS**	13.1 ± 0.5 a	19.7 ± 0.1 a	49.7 ± 0.9 a	161.7 ± 1.2 a
**W5**	**C**	11.1 ± 0.4 ab	16.6 ± 0.2 b	31.1 ± 0.3 b	99.07 ± 1.3 ab
	**T**	9.0 ± 0.4 c	14.7 ± 0.1 c	27.7 ± 0.3 c	82.47 ± 1.2 c
	**BS**	12.2 ± 0.3 a	17.6 ± 0.2 a	34.4 ± 0.6 a	105.4 ± 2.2 a
	**TBS**	10.3 ± 0.2 bc	17.2 ± 0.1 ab	32.0 ± 0.9 ab	95.80 ± 1.3 b

Trt. – treatment; C – Control; T – Triticonazole; BS – Biostimulant; TBS – Triticonazole + Biostimulant. W1–W5 – watering intervals. RDW – Root Dry Weight; SDW – Shoot Dry Weight. W1–W5 – watering intervals (W1: immediate; W2–W5: watering at 2–5 weeks after sowing). Different letters indicate statistically significant differences among seed treatments within each watering regime according to Tukey’s test ($p < 0.05$). Letters should not be compared across watering regimes.

Table S4. Nitrogen concentration and accumulation in roots and shoots of wheat (Triticum aestivum) seedlings at BBCH 12 under different seed treatments and watering regimes.

		Root N		Shoot N	
**Watering**	**Trt.**	**Conc. (%)**	**Acc. (mg plant^{-1})**	**Conc. (%)**	**Acc. (mg plant^{-1})**
**W1**	**C**	2.12 ± 0.07 a	0.99 ± 0.04 d	4.76 ± 0.02 c	6.30 ± 0.02 d
	**T**	2.19 ± 0.06 a	1.26 ± 0.03 c	5.10 ± 0.10 b	8.59 ± 0.25 c
	**BS**	2.35 ± 0.10 a	1.53 ± 0.06 b	5.27 ± 0.02 ab	9.61 ± 0.04 b
	**TBS**	2.42 ± 0.04 a	1.73 ± 0.04 a	5.50 ± 0.06 a	10.39 ± 0.14 a
**W2**	**C**	2.11 ± 0.08 b	1.08 ± 0.04 d	4.74 ± 0.01 b	6.61 ± 0.03 c
	**T**	2.19 ± 0.05 ab	1.30 ± 0.04 c	4.91 ± 0.07 b	7.49 ± 0.17 c
	**BS**	2.36 ± 0.04 a	1.82 ± 0.03 b	5.31 ± 0.04 a	11.20 ± 0.23 b
	**TBS**	2.38 ± 0.03 a	2.01 ± 0.03 a	5.51 ± 0.09 a	12.67 ± 0.33 a
**W3**	**C**	2.05 ± 0.02 b	1.09 ± 0.02 c	4.68 ± 0.03 c	7.31 ± 0.13 c
	**T**	2.13 ± 0.03 b	1.25 ± 0.01 b	4.65 ± 0.09 c	7.55 ± 0.19 c
	**BS**	2.40 ± 0.02 a	1.67 ± 0.03 a	5.15 ± 0.02 b	10.66 ± 0.10 b
	**TBS**	2.40 ± 0.01 a	1.75 ± 0.04 a	5.45 ± 0.08 a	11.71 ± 0.13 a
**W4**	**C**	2.01 ± 0.03 c	0.75 ± 0.02 c	4.61 ± 0.01 b	5.20 ± 0.18 c
	**T**	2.06 ± 0.02 c	0.75 ± 0.01 c	4.41 ± 0.10 b	4.93 ± 0.30 c
	**BS**	2.28 ± 0.02 b	1.04 ± 0.02 b	4.93 ± 0.04 a	7.29 ± 0.20 b
	**TBS**	2.41 ± 0.02 a	1.20 ± 0.01 a	5.12 ± 0.02 a	8.28 ± 0.08 a
**W5**	**C**	1.99 ± 0.11 a	0.62 ± 0.02 b	4.65 ± 0.05 ab	4.61 ± 0.10 a
	**T**	2.04 ± 0.11 a	0.56 ± 0.03 c	4.35 ± 0.14 b	3.59 ± 0.11 b
	**BS**	2.25 ± 0.12 a	0.78 ± 0.04 a	4.72 ± 0.04 ab	4.97 ± 0.14 a
	**TBS**	2.45 ± 0.10 a	0.78 ± 0.02 a	5.02 ± 0.09 a	4.81 ± 0.02 a

Trt. – treatment; C – Control; T – Triticonazole; BS – Biostimulant; TBS – Triticonazole + Biostimulant. W1–W5 – watering intervals. Conc. – concentration; Acc. – accumulation. W1–W5 – watering intervals (W1: immediate; W2–W5: watering at 2–5 weeks after sowing). Data are presented as mean ± SE. Different letters indicate statistically significant differences among seed treatments within each watering regime according to Tukey's test ($p < 0.05$). Letters should not be compared across watering regimes.
